# Single Nucleotide Polymorphism rs17849071 G/T in the *PIK3CA* Gene Is Inversely Associated with Follicular Thyroid Cancer and *PIK3CA* Amplification

**DOI:** 10.1371/journal.pone.0049192

**Published:** 2012-11-21

**Authors:** Jeffrey C. Xing, Ralph P. Tufano, Avaniyapuram Kannan Murugan, Dingxie Liu, Gary Wand, Paul W. Ladenson, Mingzhao Xing, Barry Trink

**Affiliations:** 1 Department of Otolaryngology-Head and Neck Surgery, Johns Hopkins University School of Medicine, Baltimore, Maryland, United States of America; 2 Division of Endocrinology and Metabolism, Department of Medicine, Johns Hopkins University School of Medicine, Baltimore, Maryland, United States of America; Indiana University School of Medicine, United States of America

## Abstract

The proto-oncogene *PIK3CA* has been well studied for its activating mutations and genomic amplifications but not single nucleotide polymorphism (SNP) in thyroid cancer. We investigated SNP rs17849071 (minor allele G and major allele T) in *PIK3CA* in thyroid tumors in 503 subjects by PCR and sequencing of a region of intron 9 carrying this SNP. This SNP was found in both normal and thyroid tumor tissues as well as in different generations of a studied family, confirming it to be a germline genetic event in thyroid tumor patients. In comparison with normal subjects, a dramatically lower prevalence of the heterozygous genotype G/T at rs17849071 was found in patients with follicular thyroid cancer (FTC). Specifically, rs17849071G/T was found in 15% (18/117) normal subjects vs. 1.3% (1/77) FTC patients, with an odds ratio of 0.07 (95% CI 0.01–0.55; P = 0.001). This represents a 93% risk reduction for FTC with this SNP. In contrast, no difference was seen with benign thyroid neoplasms in which the prevalence of rs17849071G/T was 13.1% (17/130), with an odds ratio of 0.83 (95% CI 0.40–1.69; P = 0.72). There was a trend of lower prevalences of rs17849071G/T and odds ratio in other types of thyroid cancer without statistical significance. We also found an interesting inverse relationship of rs17849071G/T with *PIK3CA* amplification. With copy number ≥4 defined as copy gain, 2.9% (1/34) rs17849071G/T vs. 19.0% (67/352) rs17849071T/T cases displayed *PIK3CA* amplification (P = 0.01). Conversely, 1.5% (1/68) cases with *PIK3CA* amplification vs. 10.4% (33/318) cases without *PIK3CA* amplification harbored rs17849071G/T (P = 0.01). This provides an explanation for the reciprocal relationship of rs17849071G/T with FTC, since *PIK3CA* amplification is an important oncogenic mechanism in thyroid cancer, particularly FTC. Thus, the present study uncovers an interesting phenomenon that rs17849071G/T is protective against FTC possibly through preventing *PIK3CA* amplifications.

## Introduction

Thyroid cancer is the most common endocrine malignancy, with 56,460 new cases and 1,780 related deaths estimated for 2012 and a still rapidly rising incidence in the United States [1]. Thyroid cancer can be histologically classified into papillary thyroid cancer (PTC), follicular thyroid cancer (FTC), anaplastic thyroid cancer (ATC), and medullary thyroid cancer (MTC), which account for approximately 80%, 15%, 2%, and 3% of all thyroid malignancies, respectively [2], [1]. PTC, FTC and ATC derive from follicular epithelial thyroid cells while MTC derives from parafollicular C cells. Derived from follicular epithelial thyroid cells are also the far more common benign thyroid neoplasms, including thyroid adenomas and hyperplasia. An uncommon FTC-like, but histologically and genetically distinct thyroid cancer, Hurthle-cell thyroid cancer (HTC), is also derived from follicular-epithelial thyroid cells. FTC and PTC are usually differentiated thyroid cancer. ATC is an undifferentiated and aggressive form of thyroid cancer [2].

Oncogenic genetic alterations are the driving force for the development and progression of thyroid tumors and have been extensively studied in recent years. Classical examples include *BRAF* mutation in PTC and ATC [3], [4], *Ras*, *PIK3CA* (encoding PIK3CA–the p110α catalytic subunit of phosphatidylinositol 3-kinase or PI3K), and *PTEN* mutations in benign thyroid neoplasia, FTC and ATC [5]–[8], and *RET* mutation in MTC [9]. We previously found common genetic amplifications of the *PIK3CA* gene in thyroid cancer [10], which was confirmed in several later studies [11]–[15]. The genomic copy gain of *PIK3CA* gene is functionally important as it is associated with robust expression of the PIK3CA protein in thyroid cancer [11]. PIK3CA is a key component of the PI3K/Akt signaling pathway that plays an important role in cell growth and proliferation and tumorigenesis [15]–[17] (Fresno et al, 2004; Jiang BH and Liu LZ, 2009). PI3K catalyzes the phosphorylation of the 3′-OH group of the inositol ring in inositol phospholipids, leading to production of phosphatidylinositol-3,4,5-trisphosphate [PI(3,4,5)P3] and PI(3,4)P2. Through interaction with these latter molecules in the plasma membrane of the cell, the Ser/Thr kinase Akt is translocated to plasma membrane where it becomes phosphorylated and activated by the phosphoinositide-dependent kinase. Activated Akt transduces the signaling further by phosphorylating various down-stream protein substrates, which ultimately alter expression of genes. Activating mutations and genomic amplifications of the *PIK3CA* gene have been found also in many other human cancers [18]–[20]. Therefore, through activating mutations or genomic amplification, the *PIK3CA* gene plays an important oncogenic role in human tumorigenesis. Interestingly, among differentiated thyroid tumors, *PIK3CA* copy gain occurs most commonly in FTC [10]–[12], consistent with the fact that aberrant activation of the PI3K/Akt pathway plays a particularly important role in the tumorigenesis of FTC among these tumors [7], [8].

Some single nucleotide polymorphisms (SNP) in various exons of the *PIK3CA* gene have also been found although their functional significance in human cancer is unclear [21], [10]. In the present study, we investigated the relationship of a SNP, rs17849071 (http://www.ncbi.nlm.nih.gov/SNP/snp_ref.cgi?rs=rs17849071), in the *PIK3CA* gene with various types of thyroid tumors. In contrast to all the common oncogenic genetic alterations that are associated with high cancer risk, this SNP was associated with a significantly reduced risk of development of FTC and *PIK3CA* amplification, adding a novel dimension to the genetic arrays in thyroid tumorigenesis.

## Materials and Methods

### Human tissues and DNA isolation

Human thyroid tumor tissues or blood were obtained following the approval of the institutional review board (IRB) of the Johns Hopkins University School of Medicine and genomic DNA was isolated as previously described [10], [11], [15]. Required written informed patient consents were obtained as approved by our IRB. Briefly, for DNA isolation from paraffin-embedded tissues, samples were first treated for 8-h at room temperature with xylene, followed by two additional 1-h treatments with xylene. Tissue digestion was performed with 1% SDS and 0.5 mg/ml proteinase K (Invitrogen, Carlsbad, CA) at 48°C for 48 h. To facilitate the digestion, a mid-interval addition of a spiking aliquot of 20% proteinase K was added twice a day. DNA was subsequently isolated by standard phenol-chloroform extraction and ethanol precipitation procedures. To isolate white blood cells (WBC), five ml blood was mixed with 40 ml of 20 mM Tris buffer (pH 7.0) containing 5 mM MCl_2_. After lysis of red blood cells (RBC), the mixture was centrifuged to pellet the WBC and the procedure was repeated once to completely remove RBC and hemoglobulin. The white WBC pellet was then subjected to SDS-proteinase K digestion and DNA isolation was completed as stated above.

### Analysis of rs17849071 G/T in the PIK3CA Gene by Direct DNA Sequencing

SNP rs17849071 is the 105^th^ nucleotide of intron 9 of the *PIK3CA* gene (counted downstream from the beginning of the intron), with the major allele being T and the minor allele being G. A region of the *PIK3CA* gene containing rs17849071 in intron 9 was amplified using primers GATTGGTTCTTTCCTGTCTCTG (forward) and CCACAAATATCAATTTACAACCATTG (reverse) [18] (Samuels et al, 2004). To enhance the specificity, genomic DNA was amplified using a step-down PCR protocol as follows: after an initial 3-min denaturing at 95°C, the PCR was run with each temperature for 40 sec at 6 “step-down” steps for 2 cycles each. The denaturing temperature was 95°C and extension temperature was 72°C for each of the “step-down” steps, with the annealing temperature of 66°C, 64°c, 62°c, 60°c, 58°C, and 56°C, respectively. The PCR was finally run at 95°C, 54°C, and 72°C each for 40 sec for 30 cycles, followed by a final elongation step at 72°C for 5 min. In a final volume of 25 µl, the PCR reaction mixture contained 60-80 ng genomic DNA, 67 mM Tris (pH 8.8), 6.7 mM MgCl_2,_ 16.6 mM ammonium sulfate, 10 mM 2-mercaptoethanol, 5% DMSO, 1.5 mM each dATP, dCTP, dTTP and dGTP, 1.67 µM each primers (forward and reverse), and 0.5 unit of platinum DNA Taq polymerase (Invitrogen, Carlsbad, CA). The quality of the PCR products was confirmed by electrophoresis on a 1.5% agarose gel, which consistently showed a specific single PCR product band. The PCR products were subjected to PCR reaction with Big Dye sequencing reagents (Applied Biosystems, Foster City, CA) and the sequencing primer TTGCTTTTTCTGTAAATCATCTGTG, using the following settings: 95°C for 30 sec x 1 cycle; 95°C for 15 sec, 50°C for 15 sec, and 60°C for 4 min, x 35 cycles. DNA sequencing analysis was performed for SNP identification on an ABI PRISM 3700 DNA Analyser (Applied Biosystems).

### Real-time quantitative PCR for the analysis of copy number of the *PIK3CA* gene

Detection of *PIK3CA* copy number was performed using real-time quantitative PCR and a protocol described previously [10]. Briefly, a PE Applied Biosystem ABI 7900 TaqMan sequence detector (Foster City, CA) was used with specific primers and probes designed with the Applied Biosystems software to amplify both the *PIK3CA* and control *β-actin* genes. The probe used for the *PIK3CA* gene was 5′-6-carboxyfluorescein-CACTGCACTGTTAATAACTCTCAGCAGGCAAA-tetramethylrhodamine-3′, and the primers were 5′-AAATGAAAGCTCACTCTGGATTCC-3′ (forward) and 5′-TGTGCAATTCCTATGCAATCG-3′ (reverse). For the *β-*actin gene, the probe was 5′-6-carboxyfluorescein-ATGCCCTCCCCCATGCCATCC-tetramethylrhodamine-3′, and the primers were 5′-TCACCCACACTGTGCCCATCTACGA-3′ (forward) and 5′-TCGGTGAGGATCTTCATGAGGTA-3′ (reverse). Samples were run in triplicate. Primers and probes to β-actin were run in parallel to standardize the input DNA. To establish the standard curves we used serial dilutions of DNA extracted from normal WBC cells with 0.01–20 ng DNA.

### Statistical Analysis

The odds ratio and 95% confidence intervals were calculated using standard statistical method and used to show the risk association of the heterozygous rs17849071 G/T with various types of thyroid tumors in comparison with normal control group. It reflects the chance of occurrence of rs17849071 G/T in a type of thyroid tumor in comparison with the normal control. P values for comparison with the normal control group were calculated using two-sided Fisher's exact test.

## Results

### The rs17849071 G/T or T105G Transversion Change in Intron 9 of the PIK3CA Gene Is a Germline Genetic Event

**Figure 1:**
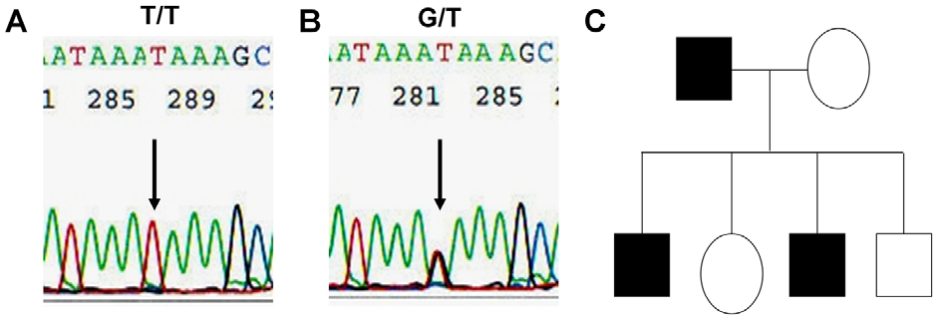
Single nucleotide polymorphism rs17849071 in intron 9 of the *PIK3CA* gene. Shown in Figure 1A is the homozygous genotype of the major allele T at rs1784907, representing the 105^th^ nucleotide of intron 9 of the *PIK3CA* gene (counting from the first nucleotide of the intron downstream). Figure 1B shows the heterozygous genotype of minor allele G and major allele T at rs17849071. Shown in Figure 1C is a family in which several members, involving two generations, harbor the heterozygous genotype rs17849071G/T (filled symbols).

The vast majority of the genotypes containing minor allele G at rs17849071 of the *PIK3CA* gene were heterozygous rs17849071G/T, as illustrated in Figure 1A and 1B. In a total of 503 subjects, only 7 cases of homozygous rs17849071G/G were found, with 1 in a benign thyroid neoplasma, 1 in HCT, and 5 in ATC. Our initial effort was to see if 17849071G, representing a transverse conversion form T, was truly a germline genetic event in thyroid tumor patients, but not a somatic alteration. In a subset of 12 cases with rs17849071G found in the primary thyroid tumors, this SNP was also found in all their matched normal thyroid tissues. Similarly, in a subset of 27 cases with no rs17849071G found in the primary tumors, this SNP was not found in any of their matched normal thyroid tissues. In four cases whose primary thyroid tumors were positive for SNP rs17849071G, the available matched WBC samples were also positive for this SNP. In a family of six members, a heterozygous rs17849071G/T was found in several of them involving two generations (filled symbols in Figure 1C). These data, in addition to showing the high frequency of rs17849071G/T in normal control group (Table 1), established that this SNP was a germline genetic event in thyroid tumor patients.

**Table 1:** Heterozygous rs17849071G/T of the *PIK3CA* Gene in Normal Subjects and Various Thyroid Tumors (Odd Ratio in comparison with normal population).

	rs17849071G/T, n/N (%)	Odds Ratio (95% CI)	P Value
Normal	18/117 (15.4%)	-	-
FTC	1/77 (1.3%)	0.07 (0.01–0.55)	0.001
PTC	10/115 (8.7%)	0.52 (0.23–1.19)	0.16
Benign	17/130 (13.1%)	0.83 (0.40–1.69)	0.72
ATC	3/38 (7.9%)	0.47 (0.13–1.70)	0.29
HTC	1/13 (7.7%)	0.46 (0.06–3.75)	0.69
MTC	2/13 (15.4%)	1.00 (0.20–4.89)	1.0
Overall	52/503 (10.3%)	-	-

### Low Occurrence of the Heterozygous Genotype G/T at rs17849071 in Follicular Thyroid Cancer

As shown in Table 1 that summarizes the occurrence of the heterozygous genotype G/T at rs17849071, this genotype was a frequent genetic event in normal control group, occurring in 18/117 (15%) normal subjects. We analyzed the relationship of this SNP with various types of thyroid tumors. Comparable frequencies of rs17849071G/T were seen in most types of thyroid tumors–9% (10/115) PTC, 13% (17/130) benign neoplasms, 8% (3/38) ATC, 8% (1/13) HTC, and 15% (2/13) MTC (Table 1). The occurrence of rs17849071G/T in these thyroid tumors was similar to that in normal population. In striking contrast, however, only 1% (1/77) FTC harbored rs17849071G/T, representing a much lower prevalence than that in normal population, with an odd ratio of 0.07 (95% CI: 0.01–0.55; p = 0.001). These data demonstrate an interesting mutual exclusion between rs17849071G/T and FTC, suggesting a protective effect of rs17849071G/T against FTC. Specifically, there was a 93% reduction in the odds of development of FTC when the heterozygous genotype G/T at rs17849071 in intron 9 of the *PIK3CA* gene exists. This SNP did not significantly affect the occurrence of other types of thyroid tumor.

### Inverse Association of rs17849071G/T with the Amplification of PIK3CA in Thyroid Tumors

**Figure 2:**
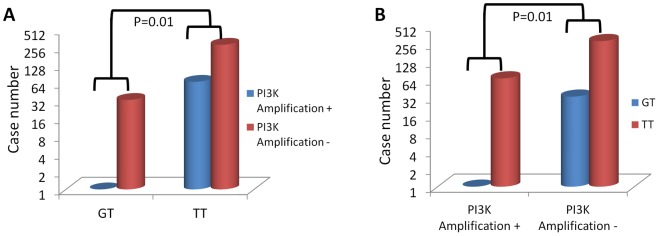
Inverse relationship of heterozygous genotype GT at rs17849071 with *PIK3CA* amplification in thyroid tumors. Figure 2A shows that in the subjects with heterozygous genotype GT at rs17849071very few cases were positive for *PIK3CA* amplification and, correspondingly, a large number of cases were negative for *PIK3CA* amplification, whereas, in contrast, a large number of subjects with homozygous genotype TT harbored *PIK3CA* amplification. Comparison of the difference between *PIK3CA* amplification-positive and -negative subjects of the GT group with that of the TT group showed a high significance (P = 0.01). Conversely, Figure 2B shows that in the subjects with *PIK3CA* amplification very few cases harbored heterozygous genotype GT at rs17849071 and, correspondingly, a large number of cases harbored homozygous TT, whereas, in contrast, a large number of subjects without *PIK3CA* amplification harbored heterozygous GT. Comparison of the difference between GT and TT subjects of the *PIK3CA* amplification-positive group with that of the *PIK3CA* amplification-negative group showed a high significance (P = 0.01).

To explore a possible mechanism for the protective effect of rs17849071G/T against FTC, we examined its relationship with various genetic alterations in thyroid tumors. We found an interesting inverse relationship of the heterozygous rs17849071G/T with genomic amplification/copy gain of the *PIK3CA* gene. Specifically, with copy number ≥4 defined as copy gain, 2.9% (1/34) rs17849071G/T vs. 19.0% (67/352) rs17849071T/T cases displayed *PIK3CA* amplification (P = 0.01). Conversely, 1.5% (1/68) cases with *PIK3CA* amplification vs. 10.4% (33/318) cases without *PIK3CA* amplification harbored heterozygous rs17849071G/T (P = 0.01). The inverse relationship between rs17849071G/T and *PIK3CA* amplification is more clearly shown in Figure 2. This exclusivity of *PIK3CA* amplification by heterozygous rs17849071G/T may explain how this SNP can protect against FTC since *PIK3CA* amplification plays a prominent role in FTC [7], [11], [12]. There was no specific relationship found for rs17849071G/T with other genetic alterations, such as mutations in *Ras*, *BRAF*, *PTEN* genes and in the *PIK3CA* gene itself (data not shown).

## Discussion

Previous genetic studies on the *PIK3CA* gene in thyroid cancer have been mostly focused on somatic oncogenic genetic alterations, such as activating mutations and genomic amplification. Little is known about the role of polymorphism of the *PIK3CA* gene in the development of thyroid cancer. The present work on rs17849071G/T represents a novel genetic study on thyroid tumors. The striking finding was the extremely low occurrence of heterozygous genotype G/T at rs17849071 in intron 9 of the *PIK3CA* gene preferentially in FTC, associated with a significantly decreased odd ratio (93% reduction) for the development of FTC. Only 1.3% (1/77) cases of FTC vs. 15% (18/117) normal subjects (P = 0.001) and the average 10.3% (52/503) of all subjects (P = 0.005) harbored the rs17849071G/T, suggesting that presence of rs17849071G/T in an individual would greatly protect against the development of FTC.

Polymorphism, particularly SNP, is common and accounts for about 90% of sequence variations in the human genome [22]. Although most SNPs seem to be functionally silent, many are associated with increased risk for the development of certain diseases or disease characteristics. Some SNPs in the coding area of a gene may affect the function of the protein product of the gene while other SNPs in regulatory regions may affect the expression of the gene [23]. This latter group of SNPs usually occur in promoters, silencers, enhancers, and introns. They may affect gene expression by altering the binding properties of regulatory regions of a gene with transcription and regulatory factors, ultimately interfering with the transcription or splicing processes.

Certain SNPs have been reported to affect thyroid cancer. For example, the L769L polymorphism of the *RET* gene seemed to affect the onset age of familial MTC [24]. Certain *RET* gene SNPs were shown to be associated with an increased risk of differentiated thyroid cancer [25], [26]. A XRCC3 18067T polymorphic allele was similarly shown to be associated with differentiated thyroid cancer [27]. More extensive genome-wide association studies have recently shown the association of several SNPs with the occurrence of thyroid cancer [28], [29]. Unlike these thyroid cancer-promoting SNPs, the rs17849071G/T found in the present study decreases the risk for FTC, which represents a rare example of tumor suppressor SNP.

Because the tumor suppressor function of the rs17849071G/T was only seen in FTC, but not other types of thyroid tumors (Table 1), it seems to be true that this SNP affects an oncogenic process that is unique and critical to the tumorigenesis of FTC. Our finding of the inverse association of the rs17849071G/T with *PIK3CA* amplification provides such a mechanism explaining the protective effect of this SNP. As *PIK3CA* amplification plays a prominent role in the tumorigenesis of thyroid cancer, particularly FTC [7], the significant decrease in the occurrence of *PIK3CA* amplification in association with rs17849071G/T may conceivably result in decreased development of FTC in the presence of this SNP. It is also possible that the rs17849071G/T may negatively affect the expression of the *PIK3CA* gene, minimizing its oncogenicity and hence reducing the development of FTC. As the rs17849071G/T is in intron 9 of the *PIK3CA* gene, or the middle of the gene, this SNP could affect the binding and function of the splicing machinery, thus limiting the production of normal mRNA of the *PIK3CA*. Interestingly, the 5 cases of homozygous rs17849071G/G in ATC all harbored *PIK3CA* amplifications. Therefore, the mutual exclusivity seems to occur only between *PIK3CA* amplification and heterozygous rs17849071G/T, but not the homozygous rs17849071G/G. However, in undifferentiated ATC, the copy gain of *PIK3CA* may not necessarily represent genomic amplification; it may represent chromosomal aneuploidy [7], thus not following the relationship of rs17849071with *PIK3CA* amplification in differentiated thyroid tumors. It remains to be elucidated how rs17849071G/T is linked to suppression of *PIK3CA* amplification. One possibility that can be speculated is that rs17849071G/T may affect the binding of DNA with an unknown regulator that plays an important role in the process of gene amplification.

The discovery of this novel FTC-suppressor SNP is interesting. If this can be confirmed in larger studies, it will add a new dimension to the currently known genetic alteration arrays in thyroid cancer and represents a novel pathway that plays a critical role in FTC tumorigenesis. Mechanistic elucidation of this phenomenon will lead to important insights into the molecular mechanisms involved in the development of FTC and to the discovery of novel therapeutic targets as well. The rs17849071G/T represents also the first genetic marker that predicts the absence of FTC with high accuracy. It may therefore be useful in excluding a diagnosis of FTC in appropriate clinical settings.
